# Antitumor efficacy of cobalt–zinc ferrite nanoparticles on MCF-7 cell line and Ehrlich ascites carcinoma bearing mice

**DOI:** 10.1038/s41598-026-54344-z

**Published:** 2026-05-28

**Authors:** Mona M. Elwan, Eman E. El-Nahass, Sabry A. El-Naggar, B. I. Salem

**Affiliations:** 1 Zoology Department, Faculty of Science, Tanta University, Tanta, 31527 Egypt; 2 Physics Department, Faculty of Science, Tanta University, Tanta, 31527 Egypt

**Keywords:** Antitumor, Cobalt/Ferrite, EAC-bearing mice, Flow cytometry, FTIR, MCF-7, Nanoparticles, SEM, Biotechnology, Cancer, Drug discovery

## Abstract

Cobalt–zinc ferrite nanoparticles (Co_1−x_Zn_x_Fe_2_O_4_; x = 0, 0.35) were synthesized using a flash-combustion method and characterized by FTIR and SEM to evaluate their structural and surface properties. This study investigated the in vitro and in vivo antitumor efficacy of cobalt ferrite nanoparticles (CF NPs). In vitro, human breast cancer cells (MCF-7) were used to determine cytotoxicity (IC_50_), apoptosis, and cell cycle effects. The IC_50_ of CF NPs was 144 µg/mL. Treatment with 1/10 IC_50_ increased necrotic, early apoptotic, and late apoptotic cell populations to 7.2%, 9.5%, and 12.6%, respectively. CF NPs arrested the cell cycle at sub-G1 and G2/M phases, while IC_50_ of cisplatin was 11.25 µg/mL. Treatment with1/10 IC_50_ arrested the cell cycle at S and G2/M phases. For in vivo analysis, forty female albino mice (CD-1) were divided into four groups. Tumor induction was performed using Ehrlich ascites carcinoma (EAC) cells (1 × 10^6^/mouse). After 24 h, mice received either cisplatin (2 mg/kg) or CF NPs (150 mg/kg; 1/10 LD_50_). After 14 days, CF NP treatment significantly reduced body weight gain, tumor volume (44%), tumor cell count (28%), and viable tumor cells (30%). Additionally, CF NPs improved hematological parameters, liver and kidney function, and restored normal histological architecture of hepatic and renal tissues. CF NPs exhibit significant antitumor activity both in vitro and in vivo by inducing apoptosis, suppressing tumor growth, and improving biochemical and histopathological parameters, supporting their potential as a promising adjunct in cancer nanotherapy.

## Introduction

World Health Organization (WHO) has reported that over 50 million people have died of cancer in the last 10 years^1^. There were 19.3 million recent cases of cancer patients and almost 10 million deaths in 2020^2^. Worldwide, approximately 28.4 million new cancer cases are projected to occur in 2040, with a 47% increase from the corresponding cases in 2020. For cancer treatment, several settings were applied, including surgical removal, chemotherapy, radiotherapy, immunotherapy, gene therapy, and recently nano therapy has been applied^3^. Among these therapies, conventional chemotherapy is considered the best treatment choice; however, it has adverse side effects on vital organs^4^, which include cyclophosphamide (CTX), cisplatin (Cis), and doxorubicin (Dox) induced nephrotoxicity, cardiotoxicity, and hepatotoxicity, respectively^5–7^. Several studies have been applied to find new, effective and safe agents to treat cancer with low toxic effects on the vital organs^8^. Nanoparticles (NPs) are used either alone or combined with chemotherapy or radiotherapy to get effective therapeutic outcomes^9^. For instance, gold and silver nanoparticles (Au and Ag NPs) have been used to treat skin, breast, and colon cancer, respectively^10,11^. Nano drug delivery system has been used recently to increase anticancer drugs’ efficacy and decrease their adverse effects^12^. For instance, nanoparticles increased the efficacy of Dox against tumors in mice^13^. Among different NPs, ferrite nanoparticles (Fe_3_O_4_) were used to treat liver and lymph nodes cancer in mice^14^. Ferrite nanoparticles (NPs) are increasingly gaining momentum for their application in the diagnosis and treatment of breast cancer^15,16^. nano-sized ferrites have attracted considerable interest owing to their low cost and high efficiency in a wide range of applications including industrial, medical, and environmental applications^17^. The distribution of metal ions in the crystal structure of Ferrite nanomaterials determines the magnetic anisotropy and superparamagnetic behavior of ferrites, which makes them suitable for use in applications such as biosensors and magnetic resonance imaging (MRI), magnetic separation of biological markers, magnetic hyperthermia, and magneto resistive biosensing^18,19^. Cobalt nanoparticles (Co NPs) were used to treat lung cancer in mice^20^. Zinc oxide nanoparticles (ZnO NPs) have antitumor effects on human liver cancer cell lines^21^. Cobalt ferrite (CoF NPs) and zinc ferrite (ZnF NPs) are involved in several biomedical applications because of biocompatibility, porosity, and its effective adsorbing properties^22–26^. It has been reported that (ZnF NPs) possess a low cytotoxicity effect on human aortic endothelial cells^27^. In addition, nickel-zinc ferrite (Ni Zn F) and iron oxide (IONs) nanoparticles showed an anticancer effect against human colon cancer HT29, breast cancer MCF-7, and liver cancer HepG-2 cells^28,29^. Till now, few reports on the role of CF NPs as an anticancer agent. Therefore, this study aims to evaluate the in vitro and in vivo anticancer efficacy of the nano-complex of CF NPs on MCF-7 and EAC- bearing mice, respectively.

## Materials and methods

### Chemicals

Cobalt-Zinc ferrite nanoparticles (CZF NPs) were prepared in the Department of Physics, Faculty of Science, Tanta University. Cisplatin (Cis-diamminedichloroplatinum II), propidium iodide (PI), Annexin, MTT 3-(4,5-dimethylthiazol-2-yl)-2,5-diphenyltetrazolium bromide) tetrazolium, MCF-7 (Michigan Cancer Foundation-7), RPMI-1640, fetal calf serum, gentamycin, DMSO, phosphate buffer saline (PBS), DAPI were purchased from Sigma Company (Egypt). Serum alanine aminotransferase (ALT), serum aspartate aminotransferase (AST), creatinine, and we purchased urea kits from Bio-diagnostic Company (Egypt).

### Cobalt–zinc ferrite nanoparticles (CZF NPs) preparation

Cobalt–zinc ferrite nanoparticles (CZF NPs) were synthesized using a flash auto-combustion approach in the Physics Department, Faculty of Science, Tanta University. Appropriate stoichiometric amounts of cobalt nitrate hexahydrate (Co(NO₃)₂·6 H₂O), ferric nitrate nonahydrate (Fe(NO₃)₃·9 H₂O), zinc nitrate hexahydrate (Zn(NO₃)₂·6 H₂O), and urea (CO(NH₂)₂) were accurately weighed and thoroughly homogenized. The prepared mixture was heated on a hot plate at 80 °C until dehydration occurred and a viscous gel was obtained. Further heating initiated a spontaneous combustion reaction, resulting in the formation of fine brown powder. The process was rapid and produced dry, uniform nanoparticle samples suitable for subsequent characterization and biological evaluation^30^.

### Characterization study

These samples were characterized by Fourier Transformer Infrared spectroscopy (FTIR) which were recorded using the infrared spectrometer, (Perkin Elmer 1430, Germany) in the range 200–4000 cm^− 1^ in the KBr medium. SEM analysis was performed in a (QUANTAFEG 250-Netherland) to visualize very small topographic details on the surface.

### Human and murine cancer cell lines

Human breast adenocarcinoma (MCF-7) cell line was obtained from National Cancer Institute (Imbaba, Egypt) and transferred the cells to the Regional Center for Mycology and Biotechnology, Al-Mansoura University, and preserved in a liquid nitrogen tank (− 170 °C) until use. MCF-7 cell line was authenticated using a standard method and were tested to detect the mycoplasma infection by using the mycoplasma kits. Murine Ehrlich ascetic carcinoma (EAC) cell line was purchased from the National Cancer Institute (Imbaba, Egypt).

**Figure:**
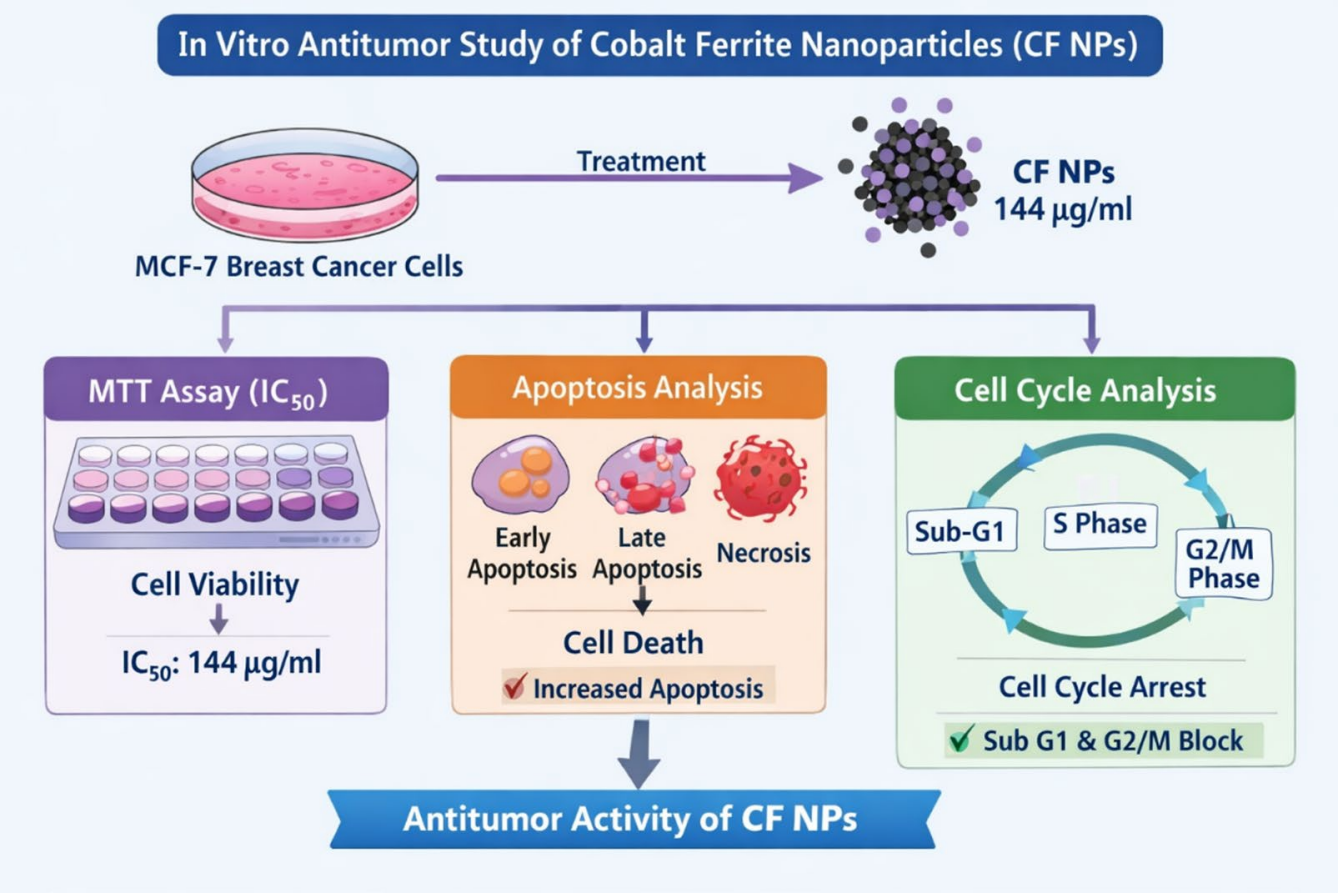
Schematic diagram shows in vitro anti-tumor study of cobalt ferrite nano particles (CF NPs).

#### MTT assay

MCF-7 cells were grown on RPMI-1640 media supplemented with 10% fetal calf serum and 50 µg/mL gentamycin at 37 °C in a humidified chamber with 5% CO_2_. For the antitumor assay, three replicas of twelve concentrations of CF NPs and reference drug Cis were suspended in 0.5% DMSO for 24 h. Optical density (OD) was measured at 590 nm after the exposure for 24 h (Sunrise, TECAN, Inc, USA). Half maximal inhibitory concentration (IC_50_) was calculated using Graphed Prism software (San Diego, CA, USA).

#### Apoptotic assay

To determine the apoptotic cells, A Fresh 1 × 10^6^ of MCF-7 cells were cultured in 6-well plates, and then we treated them with 1/10 IC_50_ of either Cis or CF NPs for 24 h. After that, treated cells were harvested and rinsed twice in PBS, followed by the binding buffer, and then 1 µL of FITC-Annexin V/ PI was added and incubated at 4 °C for 40 min. Cells were stained, then washed twice, and re-suspended in 150µL of the binding buffer with the addition of 1µL of PI (1 µg/mL). Finally, the cells were analyzed using the flow cytometer BD FACS Calibur (BD Biosciences, San Jose, CA).

#### Determination of MCF-7 cell cycle after CF NPs treatment

Cell cycle analysis was determined as described by^31^. MCF-7 cells were seeded and treated with 1/10 IC_50_ of CF NPs or Cis for 24 h. Cells were harvested and fixed overnight in 70% cold ethanol at 4 °C. After washing with ice-cold PBS, the fixed-cell pellets were collected by centrifugation and re-suspended them in PI/RNase staining buffer, then analyzed on a flow cytometer (FACSAriaTM III). Cell-cycle was calculated using CELLQUEST software (Becton Dickinson Immunocytometry Systems, San Jose, CA).

### Experimental animals and EAC-inoculation

Female albino mice (CD-1) weighing 23 ± 2 g (5-week-old) were obtained from the Animal Facility of Helwan University, Egypt. The mice were housed in the animal facility at the Faculty of Science, Tanta University, under constant temperature (25 °C ± 2 °C and 55% ± 5% relative humidity). Mice acclimatized for a week while giving them rodent food pellets and water ad libitum. The animal care committee at the Zoology Department, Faculty of Science, Tanta University, approved the experimental design prior to performing the experiments (IACUC-SCI-TU-0232). We treated the animals humanely during the entire research period. First, we collected EAC-cells from the tumor-bearing mice and then counted them using the trypan blue technique. Finally, we adjusted the number of EAC-cells as 1 × 10^6^/200-µl normal saline then inoculated it into each mouse in the experimental groups to assess the impact of the treatment with CF NPs as antitumor effects.

#### Experimental design

Forty female albino mice (CD-1) were divided into four groups (*n* = 10): Group 1 (Gp1) was the control. Gp2, Gp3 and Gp4 were inoculated with EAC-cells as 1 × 10^6^/mouse at day-0. After 24 h of EAC-inoculation, Gp3 and Gp4 were injected intraperitoneally (i.p) with Cis (2 mg/Kg) or with 1/10 LD_50_ of CF NPs (150 mg/Kg), respectively. After 14 days of EAC-inoculation, all mice groups were sacrificed to determine the tumor indices, hematological, biochemical, and histological investigations.

#### Determination of the percentage of body weight changes

Mice were weighed at the beginning (initial b.wt) and the end of the experiments (final b.wt). The percentage of b.wt change was calculated as follows: % b.wt change = [(final b.wt − initial b.wt)/initial b.wt] × 100.

#### Determination of the hematological and biochemical analysis

Blood samples were collected from the orbital venous plexus of the mice for hematological assessment. Red blood cell count (R.B.Cs), hemoglobin concentration (Hb g/dl), total white blood cell count (W.B.Cs), and differential leukocyte count were determined using an automated hematology analyzer (BC3200, Mindray, China). For biochemical analysis, blood samples were centrifuged at 3000 rpm for 15 min to separate serum. Serum alanine aminotransferase (ALT) and aspartate aminotransferase (AST) activities were measured using commercially available diagnostic kits. Serum creatinine concentration was estimated using the kinetic method^32^, while urea levels were quantified following standard colorimetric procedures according to the manufacturers’ instructions^33^.

#### Histological investigations

Tissue sections of the liver and kidney were immediately collected after sacrificing the animals under isoflurane anesthesia. Harvested organs were sliced into small pieces and fixed in 10% formalin for 24 h. After washing to remove the excess of fixative, the tissue samples were dehydrated in ascending serial ethanol, cleared by xylene, and then embedded in paraffin wax. Sections of 5 μm thickness were mounted and stained with haematoxylin and eosin (H&E) method for histological examination^34^.

### Statistical analysis

Data were presented as mean ± SD and were analyzed using one–way analysis of variance (ANOVA) followed by Tukey’s post hoc test, and *p* < 0.05 or *p* < 0.01 were statistically significant.

### Ethics statement

This study was conducted in accordance with the ARRIVE guidelines for the ethical treatment, care, and use of animals in research. The experimental design, animal handling, and data collection procedures were carefully implemented to ensure reproducibility, transparency, and minimal animal suffering. Approval for the study was obtained from the international laboratory Animal Care and Use guidelines with ethical approval number (IAUCUC-SCI-TU-0232), and all procedures adhered to the appropriate ethical standards.

## Results

### FTIR Spectra

Fourier transform infrared (FTIR) spectroscopy was applied to examine the structural features and confirm the formation of the spinel phase in the synthesized cobalt–zinc ferrite nanoparticles. This method provides information about metal–oxygen bonding and the distribution of cations within tetrahedral and octahedral sites of the crystal lattice.

**Fig. 1:**
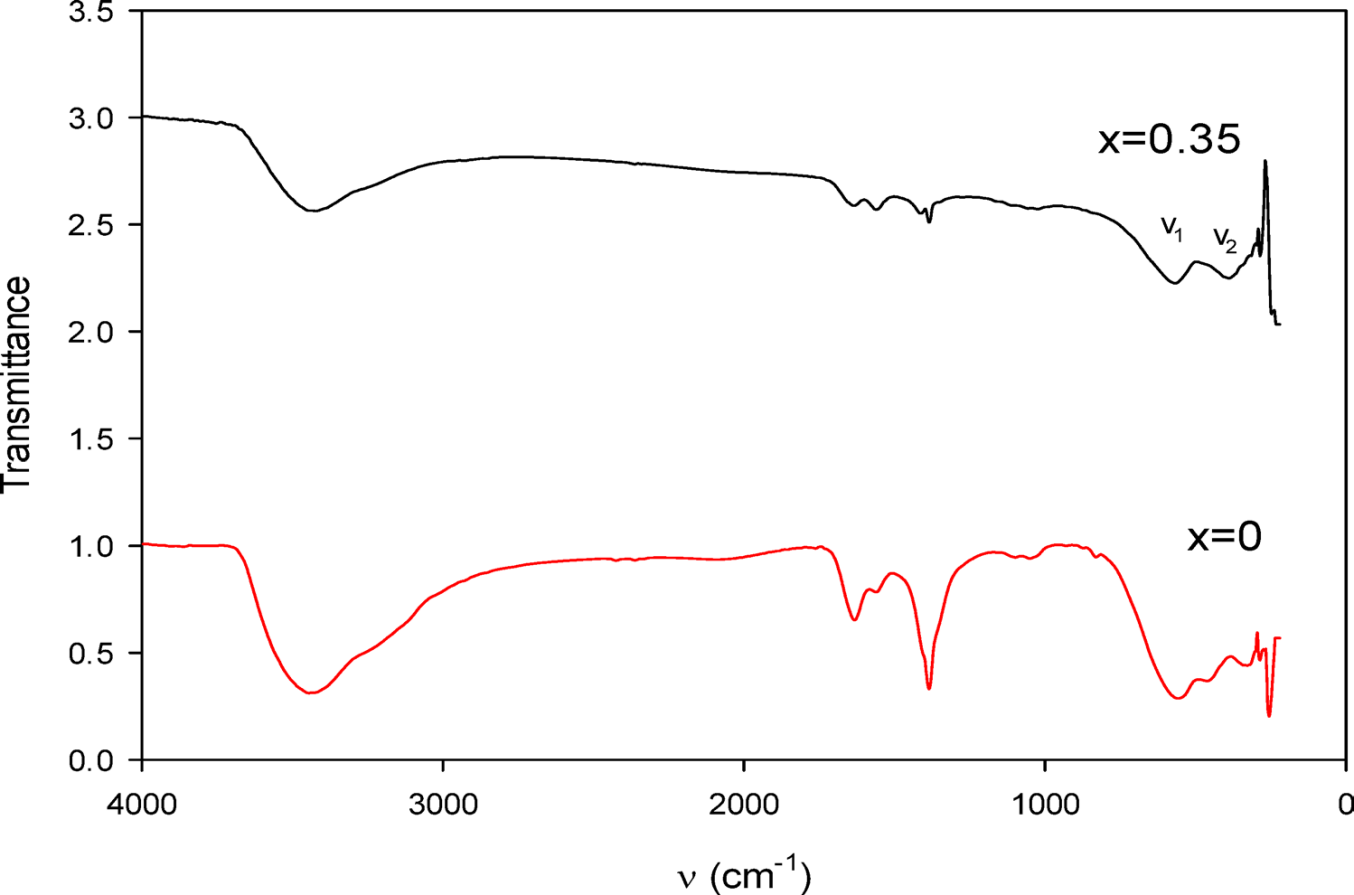
FTIR spectrum of Co_1−x_Zn_x_Fe_2_O_4_ (x = 0,0.35) nanoparticles in a wave-number range between 250 and 4000 cm^− 1^.

The FTIR spectra recorded in the range of 250–4000 cm^−1^ revealed two prominent absorption bands located between 300 and 600 cm^−1^^35^ as shown in Fig. 1a, b, which are characteristic of spinel ferrite materials. The higher-frequency band (ν_1_) is attributed to stretching vibrations of Fe–O bonds in tetrahedral positions, whereas the lower-frequency band (ν_2_) corresponds to metal–oxygen vibrations in octahedral sites involving Co^2+^ and Zn^2+^ ions^36^.

For CoFe_2_O_4_ (x = 0), these bands appeared near 570 cm^−1^ and 420 cm^−1^, respectively. In the Zn-substituted sample Co_0.65_Zn_0.35_Fe_2_O_4_ (x = 0.35), a shift of the bands toward lower wavenumbers (approximately 550 cm^−1^ and 400 cm^−1^) was observed, indicating successful incorporation of Zn^2+^ ions into the spinel lattice.

Broad bands detected around 3400 cm^−1^ and peaks near 1630 cm^−1^ were assigned to O–H stretching and bending vibrations of adsorbed water molecules. Additional minor peaks between 1000 and 1500 cm^−1^ may be related to residual nitrate groups or traces of organic species originating from the synthesis procedure.

### Scanning electron microscopy (SEM) and EDAX analysis

**Fig. 2:**
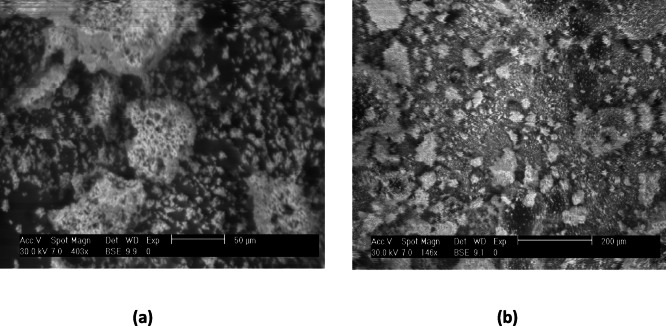
SEM microstructures of (**a**) CoFe_2_O_4_, (**b**) Co_0.65_Zn_0.35_Fe_2_O_4_ nanoparticles.

**Fig. 3:**
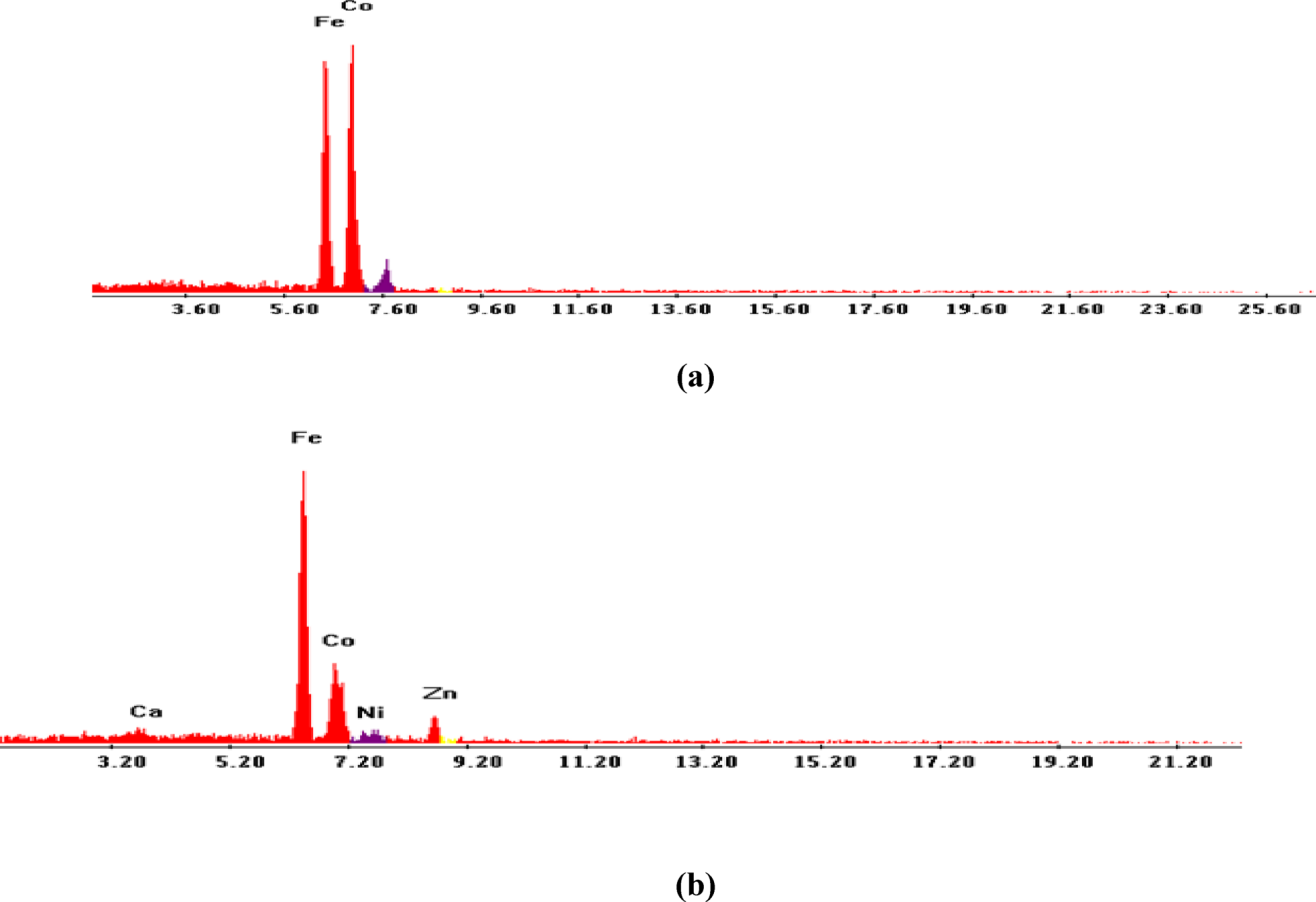
EDAX microstructures of (**a**) CoFe_2_O_4_, (**b**) Co_0.65_Zn_0.35_Fe_2_O_4_ nanoparticles.

Scanning electron microscopy (SEM) is a crucial technique for assessing the surface morphology, particle size, and agglomeration state of CoZn-ferrite nanoparticles. The SEM photographs of the cobalt ferrite magnetic nanoparticles are shown in Fig. 2a, b. The prepared nanoparticles were agglomerated with homogeneous distribution and are nearly spherical in shape. The average sizes of the particles are from 0.5 to 0.7 μm as shown in Table 1. The SEM image provides that the produced cobalt ferrites are at micro-range with brisk pores. Using the EDAX analysis, the compositions of elements present in the sample were measured. The spectrum of EDAX is given on Fig. 3a, b.

**Table 1:** Grain size of Co_1−x_Zn_x_Fe_2_O_4_ (x = 0,0.35) nanoparticles.

X	Grain size (µm)
0	0.53
0.35	0.68

### IC_50_ of CF NPs on MCF-7 cells after 24 h of treatment in vitro

**Fig. 4:**
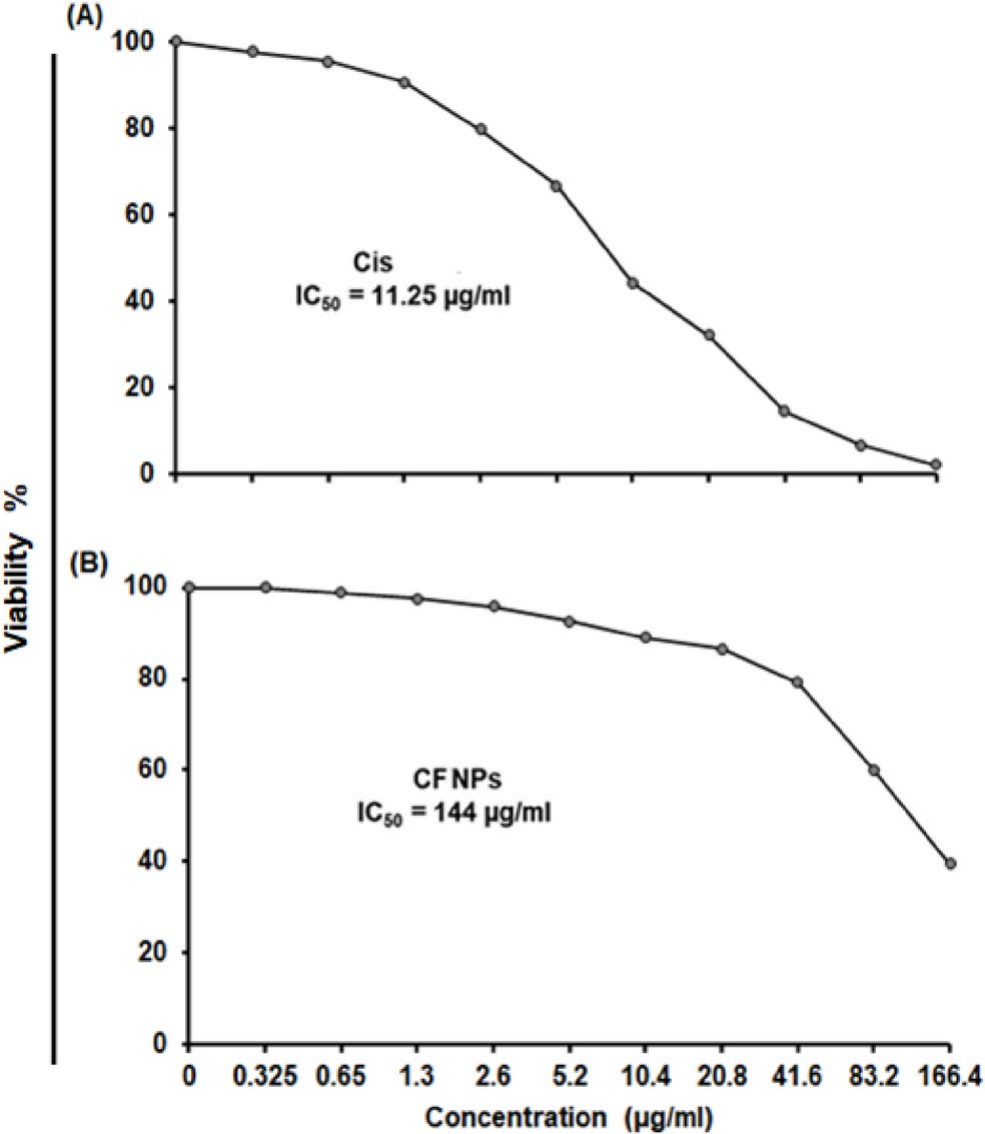
IC50 of Cisplatin (Cis) (**A**), and CF NPs (**B**) post 24 h, of exposure in vitro.

The results showed that the treatment with different concentrations of Cis (reference drug) varied from 0.325 to 166.4 µg/ml increased the inhibition percentage of MCF-7 cells in vitro in a dose-dependent manner. The median inhibition value (IC_50_) of Cis against MCF-7 cells in vitro was 11.25 µg/ml after 24 h. of exposure. However, treatment with different concentrations of CF NPs increased the inhibitory percentages of MCF-7 cells in vitro, but not much as in Cis is done. The IC_50_ of CF NPs against MCF-7 cells in vitro was 144 µg/ml after 24 h of exposure (Fig. 4).

### Treatment with CF NPs increased early and late apoptosis in MCF-7 cells in vitro

**Fig. 5:**
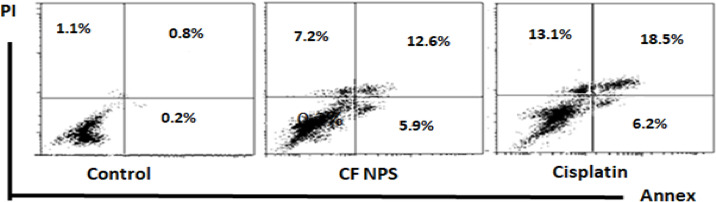
The percentages of necrotic, early, and late apoptotic MCF-7 cells after treatment with CF NPs or Cis for 24 h in vitro. Annexin V/PI double-staining assay of MCF-7 cells treated with CF NPS. The Y-axis represents the PI- labeled population, whereas the X-axis represents the FITC-labeled Annexin V positive cells. The lower left portion of the fluorocytogram (An, PI) shows normal cells, whereas the lower right portion of the fluorocytogram (An+, PI) shows early apoptotic cells. The upper right portion of the fluorocytogram (An+, PI+) shows late poptotic cells.

The data showed that the percentages of necrotic, early, and late apoptotic cells were 1.1, 0.2, and 0.8%, respectively, in the untreated MCF-7 cells after 24 h of culture. The treatment of MCF-7 cells with 1/10 IC_50_ of Cis led to a significant increase in the % of necrotic, early, and late apoptotic cells post 24 h of in vitro exposure, as represented by 13.1, 6.2, and 18.5%, respectively. The treatment with 1/10 of IC_50_ of CF NPs for 24 h increased the % of necrotic, early, and late apoptotic cells when compared with the untreated MCF-7 cells and represented by 7.2. 9.5 and 12.6% respectively, (Fig. 5).

### CF NPs treatment arrested MCF-7 cell cycle at sub G1 and G2/M phases in vitro

**Fig. 6:**
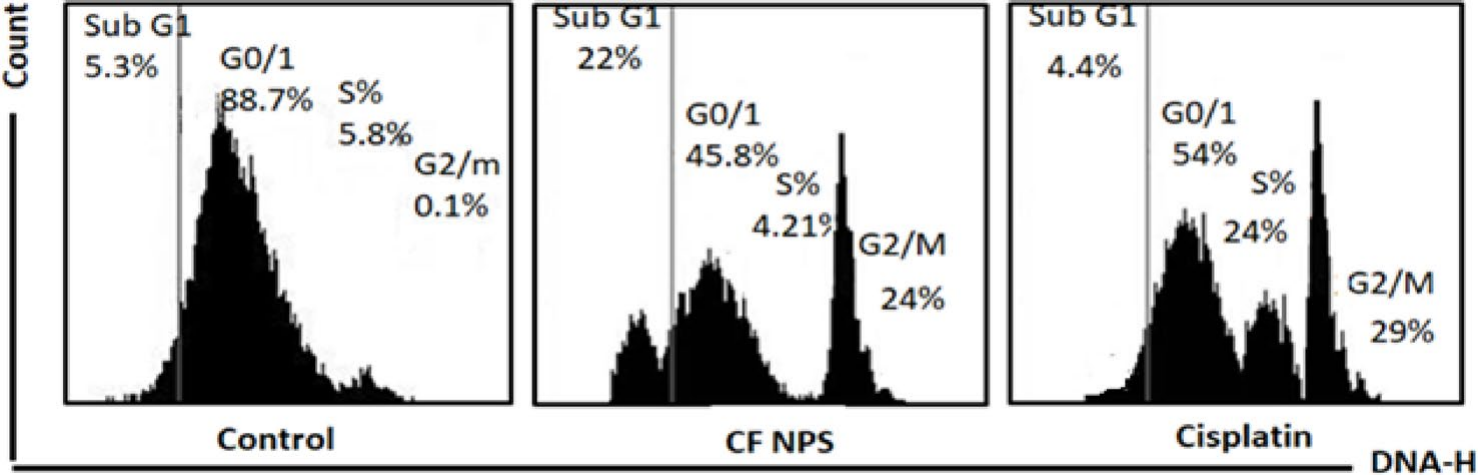
CF NPS induces cell cycle arrest in MCF-7 cells at sub G1 and G2 phases post 24 h of in vitro treatment. Cells were treated with DMSO (negative control), CF NPS (positive control). Flow cytometric analysis was performed for cell-cycle distribution. The DNA content was evaluated with propidium iodide (PI) staining and fluorescence measured and analysed. Figure 3: Representative flow cytometry graph for each treated or untreated groups.

The percentages of sub G1, S, and G2/M phases were 5.3, 88.7, 5.8, and 0.1%, respectively, in untreated MCF-7 cells. However, treatment with 1/10 IC50 of Cis arrested MCF-7 cells in S and G2/M phases. The S and G2/M phases after Cis exposure were 24 and 29%, respectively. Treatment of MCF-7 cells with 1/10 IC_50_ CF NPs for 24 h. in vitro, arrested cells at sub G1 and G2/M phases. These phases after treatment with CF NPs were 22 and 24%, respectively (Fig. 6).

### Treatment with CF NPs decreased the total body weight of mice

**Fig. 7:**
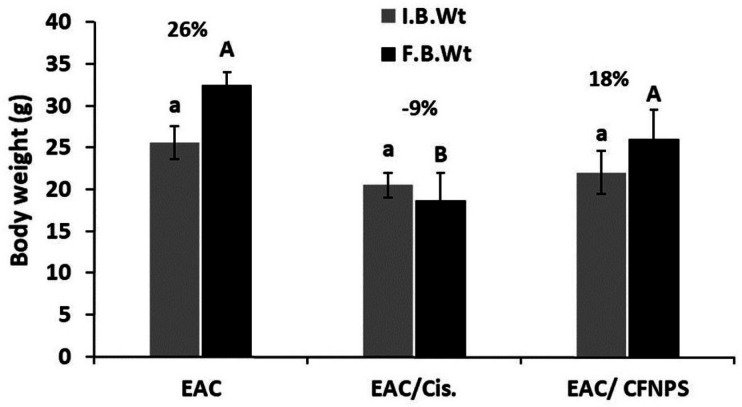
The total body weight change (T.B.W) in all groups under study.

The results showed a significant increase in the % b.wt of EAC-bearing mice compared with the control group. However, the % b.wt change decreased significantly in the EAC-bearing mice treated with Cis compared with EAC-bearing mice. In addition, the % b.wt change decreased significantly in the EAC-bearing mice treated with CF NPs compared with EAC-bearing mice alone (Fig. 7) and Table 2.

**Table 2:** Initial and final body weight of different groups under the study.

Groups	I.B.Wt	F.B.Wt	% Change
EAC alone	25.6 ± 2.0	32.5 ± 1.5	26
EAC/Cis	20.5 ± 1.5	18.7 ± 3.3	− 9
EAC/ CF NPS	22 ± 2.6	26 ± 3.5	18

The values represented mean ± SD. EAC: ehrlich ascites carcinoma, EAC/Cis: ehrlich ascites carcinoma/cisplatin, EAC/ CF NPs: ehrlich ascites carcinoma mice/cobalt ferrite nanoparticles.

### Treatment with CF NPs restored the hematological parameters close to their normal values

The results showed a significant decrease in the total R.B.C.s count, Hb concentration, and an increase in the total W.B.C.s count in EAC-bearing mice. While the treatment of EAC-bearing mice with either Cis or CF NPs restored these values close to their normal values. The platelets count decreased in EAC-bearing mice. However, after treatment with Cis or CF, NPs restored close to their normal values (Table 3). There was a significant increase in the total number of lymphocytes and monocytes and a decrease in the total number of neutrophils after the treatment of EAC-bearing mice with Cis or CF NPs compared with their volumes in EAC-bearing mice alone (Table 4).

**Table 3:** The hematological parameters in different groups of mice.

Groups	*R*.B.Cs (×10^6^/µL)	Hb (g/dL)	W.B.Cs (×10^3^/µL)	Platelets (×10^3^/µL)
EAC alone	5.15 ± 0.8 ^b^	7.8 ± 0.8 ^b^	9.5 ± 0.3 ^a^	238.7 ± 65.2 ^b^
EAC/Cis	6.91 ± 0.3 ^a^	9.5 ± 0.2 ^a^	6.9 ± 1.6 ^b^	410 ± 37.6 ^a^
EAC/CF NPS	6.57 ± 0.9 ^a^	8.3 ± 1.8 ^a^	8 ± 3.2 ^b^	363.7 ± 15.3 ^a^

The values represented mean ± SD. EAC: ehrlich ascites carcinoma, EAC/Cis: ehrlich ascites carcinoma /Cisplatin, EAC/CF NPs: ehrlich ascites carcinoma mice/cobalt ferrite nanoparticles.

**Table 4:** Absolute numbers of the differential leucocytes in different groups under the study.

Groups	Total number of different leukocytes (×10^3/^ µL)		
	Neutrophils (×10^3^/µL)	Lymphocytes (×10^3^/µL)	Monocytes (×10^3^/µL)
EAC alone	36.7 ± 10.8 ^a^	48 ± 5.5 ^a^	9 ± 10.5 ^b^
EAC/Cis	25 ± 9.6 ^b^	54 ± 16.9 ^b^	20 ± 7.2 ^a^
EAC/ CF NPS	17 ± 16.7 ^b^	55 ± 29.8 ^b^	28 ± 12.6 ^a^

The values represented mean ± SD. EAC: Ehrlich ascites carcinoma, EAC/Cis: Ehrlich ascites carcinoma /Cisplatin, EAC/ CF NPs: Ehrlich ascites carcinoma mice/ Cobalt Ferrite Nanoparticles.

### Treatment with CF NPs improved of liver and kidney functions

The results showed that the levels of ALT and AST were increased in EAC-bearing mice. But these transaminases were decreased in EAC-bearing mice treated with Cis or CF NPs compared with EAC-bearing mice only. In addition, urea and creatinine levels were increased significantly in EAC-bearing mice. While EAC-bearing mice were treated with Cis or CF, NPs showed enhanced urea and creatinine levels compared with those in the EAC-bearing group (Table 5).

**Table 5:** AST, ALT, Urea and Creatinine levels in different groups of mice.

Groups	AST (U/l)	ALT (U/l)	Urea (mg/dl)	Creatinine (mg/dl)
EAC alone	159.3 ± 1.46^a^	158.7 ± 2.37 ^a^	86.3 ± 0.5^a^	1.85 ± 0.02^a^
EAC/Cis	98 ± 2.05 ^c^	134.7 ± 1.16 ^c^	48.7 ± 4.04 ^c^	0.77 ± 0.06 ^c^
EAC/ CF NPS	132.7 ± 1.15 ^b^	146.3 ± 2.15 ^b^	55.4±0.39 ^b^	1.4 ± 0.02 ^b^

The values represented mean ± SD. EAC: Ehrlich ascites carcinoma, EAC/Cis: Ehrlich ascites carcinoma /Cisplatin, EAC/ CF NPS: Ehrlich ascites carcinoma mice/ Cobalt Ferrite Nanoparticles.

### Treatment with CF NPs alone showed an antitumor effect in vivo

**Fig. 8:**
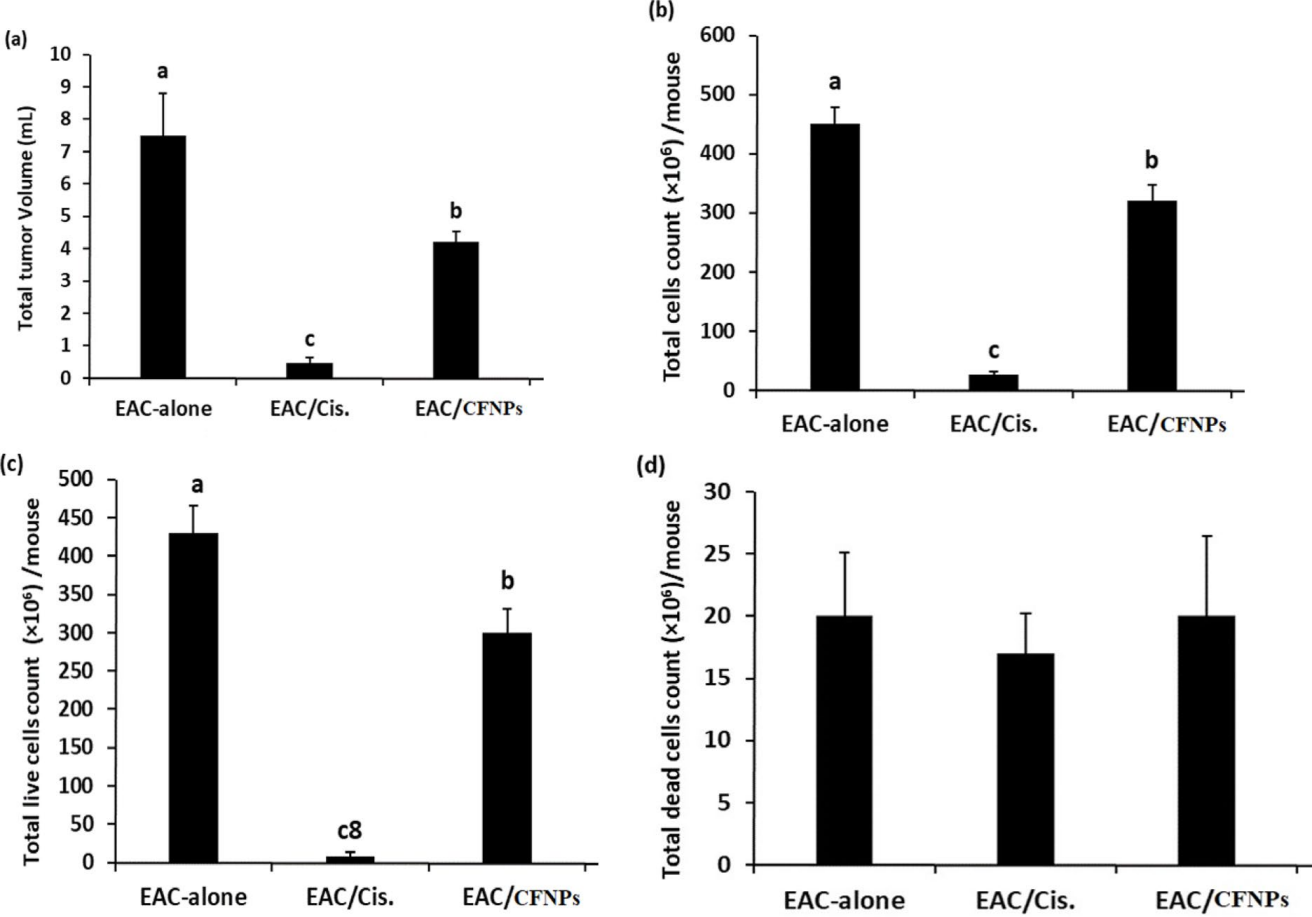
Treatment with CF NPs alone showed antitumor effect in vivo.

To assess the antitumor effect of CF NPs on EAC-bearing mice, the total tumor volumes, total live and dead tumor cells were detected. EAC-bearing mice treated with Cis alone showed a significant decrease in the total tumor volume and counts compared with EAC only. In addition, EAC-bearing mice treated with CF NPs showed a significant decrease in the total tumor volume and tumor counts compared with the EAC-bearing mice alone (Fig. 8) and Table 6.

**Table 6:** The tumor volume, tumor cells count, live and dead cancer cells in different group mice under the study.

Groups	T.V. (mL)		T.C.C (×10^6^)		T.L.C (×10^6^)		T.D.C (×10^6^)	
	M ± SD	*r*%	M ± SD	*r*%	M ± SD	*r*%	M ± SD	*r*%
EAC-alone	7.5 ± 1.3^c^	–	450 ± 29^a^	**–**	430 ± 35^a^	–	20 ± 5.1^a^	–
EAC/Cis.	0.45± 0.19^a^	94%	25 ± 6.9^c^	94%	8.0 ± 6.0^c^	98%	17 ± 3.2^a^	15%
EAC/ CF NPS	4.2 ± 0.32^b^	44%	320± 27.5^b^	28%	300 ± 31^b^	30%	20 ± 6.5^a^	5%

Gp1: EAC alone, Gp2: EAC/Cis. (2 mg/kg/6 days); Gp3: EAC/CF NPs (50 mg/Kg/6 days), r%: the percentage of reduction, T.V.: Total tumor volume, T.C.C.: Total tumor cell count, T.L.C.: Total live cells, T.D.C.: Total dead cells.

### Liver and kidney histopathological examinations

**Fig. 9:**
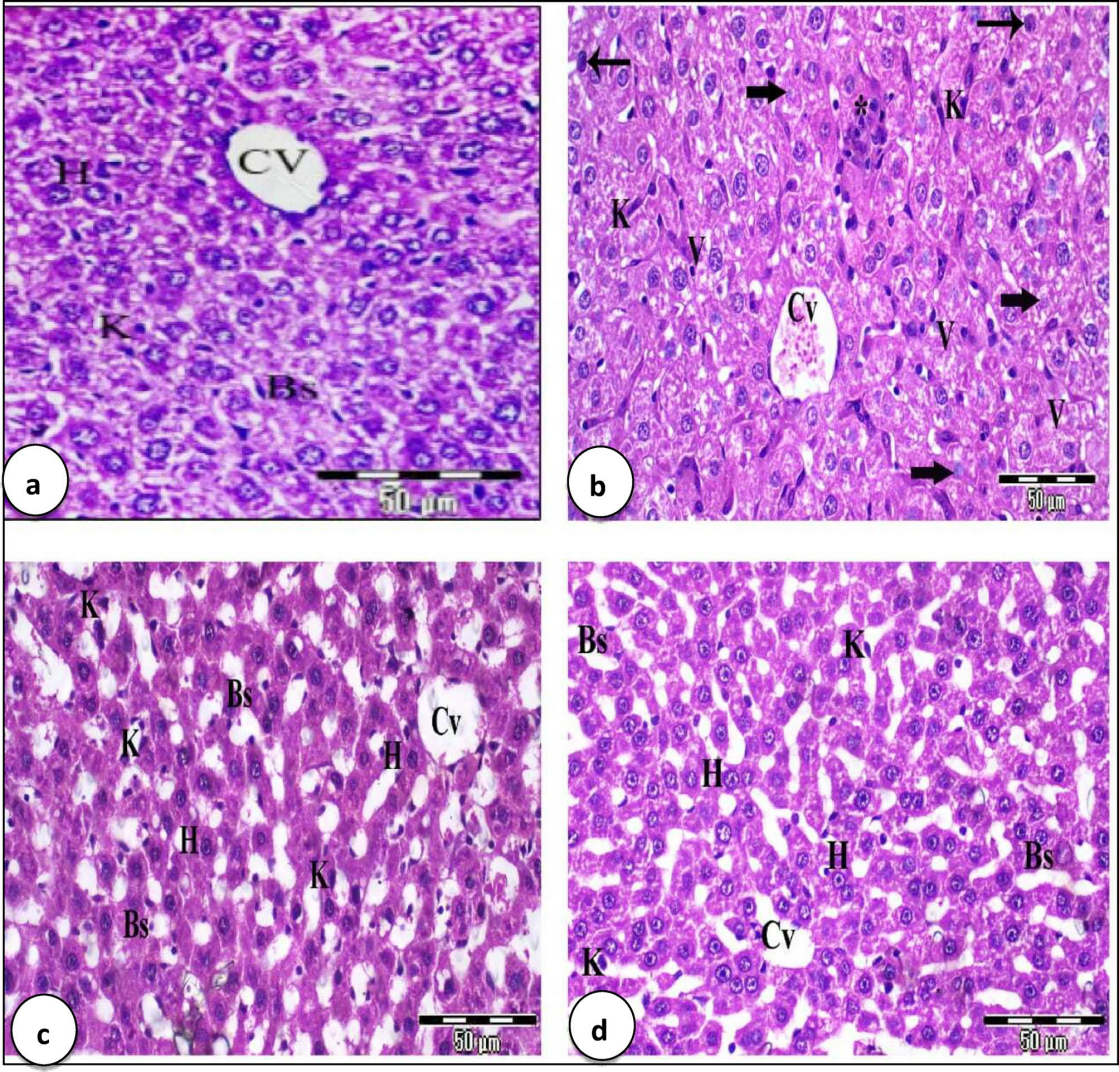
Photomicrographs of liver sections stained with H&E showing (X 400). (**a**) The liver section of control group of mice (G1) exhibits normal hepatic structure, central vein (Cv), radial hepatic strands (H), and regular blood sinusoids (Bs) with normal kupffer cells (K). (**b**) Liver sections of mice of EAC group showing dilated congested central vein (Cv) (*), disorganization of the hepatic architecture, mostly hepatocytes are vacuolated (V), others with pyknotic nuclei (arrows) and karyolitic ones (thick arrows). Also irregular blood sinusoids (Bs) with distinct Kupffer cell (K) and few mononuclear infiltration was noticed (*). (**c**) Liver sections of EAC/Cis group exhibit normal central vein (Cv), irregular strands of hepatocytes, dilated blood sinusoids (Bs) with activated Kupffer ells (K). (**d**) Liver sections of EAC/CF NPs group showing normal central veins (Cvs), normal radiating hepatocytes (H) with normal nuclei and irregular blood sinusoids (Bs) with few no of phagocytic Kupffer cells (K).

Examination of the control group (Gp1) showed that hepatocytes were radiating from the central vein. The liver strands were alternating with narrow blood sinusoids lined by endothelial cells and normal Kupffer cells (Fig. 9a). Liver sections of EAC-bearing mice showed dilated congested central vein, aggregation of mononuclear infiltration, marked disorganization of the hepatic architecture. Most hepatocytes are vacuolated, while others with pyknotic and karyolitic nuclei. There were irregular blood sinusoids with distinct Kupffer cells (Fig. 9b). Liver sections of EAC-bearing mice treated with Cis exhibited normal central vein, irregular strands of hepatocytes with normal stainability. Dilated blood sinusoids with activated Kupffer cells were observed (Fig. 9c). The liver sections of the EAC/CF NPs group showed improvement in the hepatic architecture with mostly normal central veins, normal radiating hepatocytes with normal nuclei, but blood sinusoids are irregular with few numbers of phagocytic Kupffer cells (Fig. 9d).

**Fig. 10:**
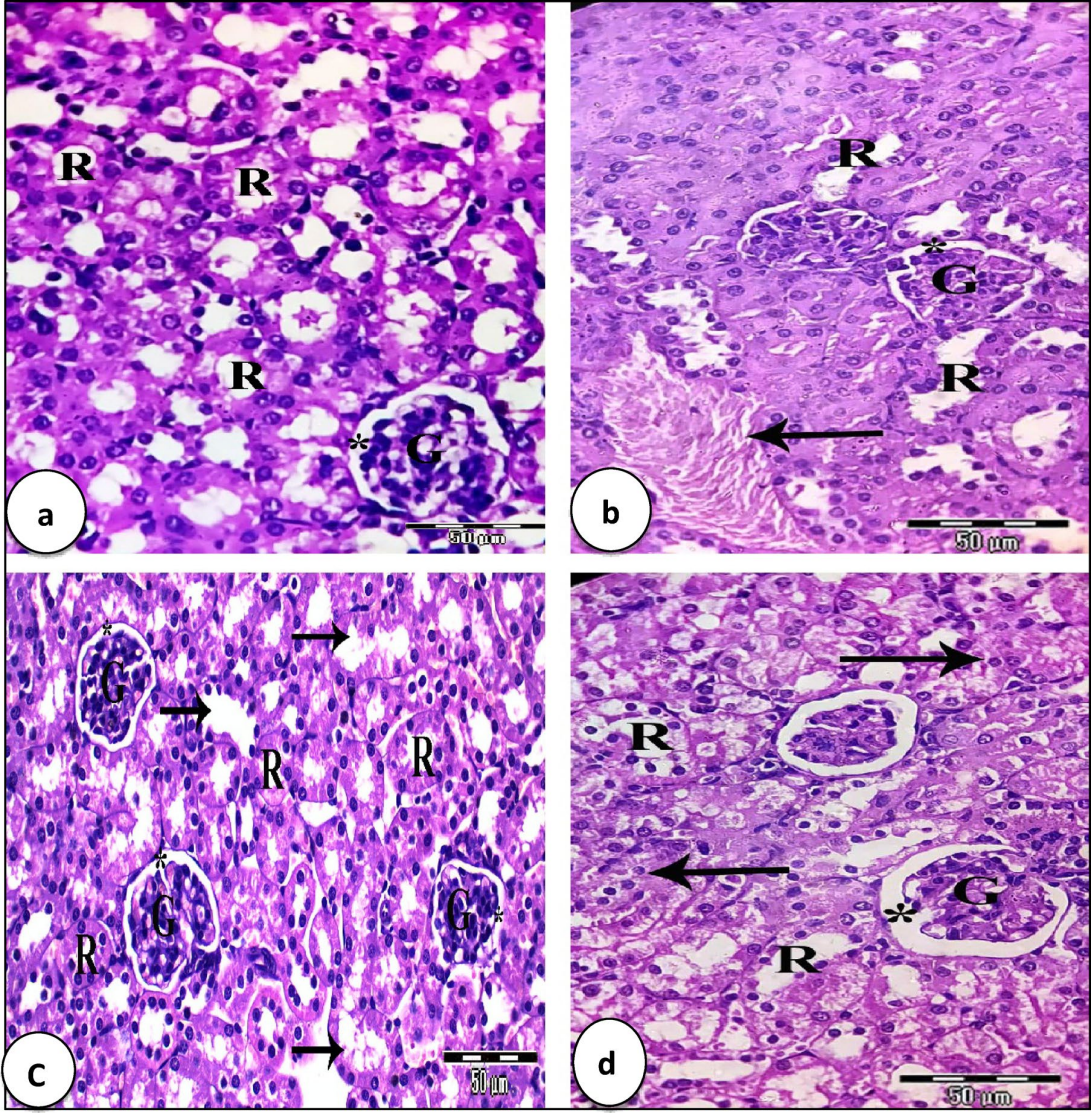
Photomicrographs of kidney sections stained with H&E (X 400). (**a**) Kidney section of the control group of mice (G1) reveals normal glomeruli (G) with normal Bowman´s space (*) and regular renal tubules (R). (**b**) Kidney sections of EAC group showing disorganized glomeruli (G) with irregular Bowman’s space (*), few numbers of renal tubules are normal (R), others are intermixed with each other’s and congested renal blood vessels were noticed (arrow). (**c**) Kidney sections of EAC/Cis group exhibits normal like glomeruli (G) with regular Bowman´s space (*), mostly renal tubules are destroyed and destructed (arrows), few ones are normal (R). (**d**) Kidney sections of mice of EAC/CF NPs exhibits organized glomeruli with regular Bowman’s space (*), mostly renal tubules are normal (R), few ones are destroyed (arrows).

Normal histological structure of the kidney tissue of the control group (Gp1) exhibited renal parenchyma that appeared as the cortex region, normal glomeruli with normal Bowman’s corpuscle, and normal renal tubules (Fig. 10a). On the contrary, kidney sections of the EAC group showed disorganized glomeruli with irregular Bowman’s space, few numbers of renal tubules were normal, others were intermixed with each and lost their characteristic appearance. Also, congested dilated renal blood vessels were noticed (Fig. 10b). Kidney sections of EAC-bearing mice treated with Cis exhibited normal glomeruli with regular Bowman’s space; mostly renal tubules were destroyed and destructed (Fig. 10c). While kidney sections of EAC-bearing mice treated with CF NPs exhibited normal like glomeruli with regular normal Bowman’s space, mostly renal tubules are normal, others are destroyed, destructed, and lost their lining epithelia (Fig. 10d).

## Discussion

The FTIR spectra confirms the successful synthesis of spinel ferrite for both compositions. The key to this confirmation lies in the presence of two main absorption bands below 1000 cm^−1^, which are characteristic of metal-oxygen stretching vibrations within the spinel lattice. The higher frequency band, ν_1_, typically in the range of 550–600 cm^−1^, corresponds to the stretching vibrations of metal-oxygen bonds in the tetrahedral (A) sites. The lower frequency band, ν_2_, usually between 400 and 450 cm^−1^, is attributed to the stretching vibrations of metal-oxygen bonds in the octahedral (B) sites. For CoFe_2_O_4_ (x = 0), the ν_1_ band at 570 cm^−1^ and ν_2_ band at 420 cm^−1^ are consistent with reported values for cobalt ferrite^37–39^, Upon zinc substitution in Co_0.65_Zn_0.35_Fe_2_O_4_ (x = 0.35), a significant observation is the shift of the ν_1_ band from 570 cm^−1^ (for x = 0) to 550 cm^−1^ (for x = 0.35). This downward shift in wavenumber for the tetrahedral site vibration is a strong indicator of successful Zn^2+^ incorporation into the tetrahedral sites. The ν_2_ band also shows a slight shift to a lower wavenumber (from 420 to 400 cm^−1^), which can be attributed to the redistribution of Fe^3+^ and Co^2+^ ions within the octahedral sites because of Zn^2+^ preferring tetrahedral sites. Absorption bands observed within the limit 300–600 cm^− 1^ reveal the formation of single-phase spinel structure having two sub-lattices, tetrahedral (A) site and octahedral (B) site^40,41^ which agree with XRD results^42^.

The broad absorption bands around 3400 cm^−1^ and the peak at 1630 cm^−1^ in both spectra are due to the O–H stretching and bending vibrations of adsorbed water molecules, respectively^39,43^. As nitrate precursors were used as the starting material, the bands near 1380 cm^− 1^ reveal N–O stretching vibrations arising from the nitrates group^44,45^. The additional sharper peaks between 1000 and 1500 cm^−1^ in the x = 0 sample, but less so in x = 0.35, could suggest residual organic precursors or minor impurities from the synthesis method that are more prevalent in the pure cobalt ferrite, or perhaps indicate differences in surface functionalization.

SEM images reveal the microscopic structure and morphology of CoFe_2_O_4_ and Zn-substituted Co_0.65_Zn_0.35_Fe_2_O_4_ nanoparticles as shown in Fig. 2. The micrograph of pure CoFe_2_O_4_ (Fig. 2a) shows dense agglomerations of fine particles with spherical morphology and rough surfaces. The strong tendency for agglomeration can be attributed to the magnetic dipole–dipole interactions among nanoparticles as well as their high surface energy. In contrast, the Co_0.65_Zn_0.35_Fe_2_O_4_ sample (Fig. 2b) exhibits a more loosely packed structure with relatively larger grains and a more uniform distribution. The reduced agglomeration and smoother spherical surface morphology are likely a result of Zn^2+^ incorporation into the spinel lattice, which modifies the crystal growth process and decreases the overall magnetic interactions between particles which agree with^42^.

Treatment with chemotherapies causes several side effects on the body’s vital organs. Besides the conventional therapies, different nanoparticles were used in several pre-clinical studies as anticancer agents^46^. This study was conducted to evaluate the in vitro and in vivo anticancer efficacy of CF NPs. CF NPs showed potential cytotoxicity against human breast cancer (MCF-7) cell lines in this study. The in vitro study showed that the treatment of MCF-7 cells with 1/10 IC_50_ of CF NPs led to a marked increase in the percentages of necrotic, early, and late apoptotic MCF-7cells. This finding could be because of the toxic effect of this nano-complex on the MCF-7 cells by initiating the extrinsic or the intrinsic apoptotic pathways. This result agreed with previous studies that showed that exposure of human colon cancer HT29, breast cancer MCF-7, and liver cancer HepG-2 cell lines to nickel-zinc ferrite (Ni Zn F) and iron oxide (IONs) nanoparticles in vitro led to a significant increase in necrotic, early apoptotic and late apoptotic cells^28,29^. Treatment with 1/10 IC_50_ of Cis arrested MCF-7 cell cycle in S and G2/M phases, while the treatment with 1/10 IC_50_ CF NPs arrested these cells at sub G1 and G2/M phases. Consistent with these findings^47^, reported that ZnO NPs could arrest MCF-7 cells at sub G1 phase. Treatment of EAC-bearing mice with CF NPs for 14 days decreased the % b.wt change compared with their values in the group of mice that inoculated with EAC-cells alone. This result was in line with a previous study by^48^, who found that % b.wt change decreased after the treatment with ZnO NPs. The treatment with CF NPs showed a decrease in the total tumor volume, total tumor counts of live and dead cells, in agreement with this result^49^, reported that CF NPs had an antitumor effect against colon tumor cells.

Compared with EAC-bearing mice alone, the total number of R.B.Cs and Hb were increased after the treatment with either Cis or CF NPs. This improvement could be because of the elimination of the stress that induced by the presence and proliferation of EAC-cells for 14 days. Previous studies showed the treatment with Au NPs improved the hematological parameters in mice^50^. The result showed a significant increase in the total W.B.Cs count in EAC-bearing mice. Mice injected with Cis, or CF NPs showed a significant decrease in W.B.Cs count; this finding was in line with a previous study that showed the total W.B.Cs count decreased post 3 days of chemotherapeutic injection^51^. In addition, they showed that the leucopenic phase post chemotherapeutic injection extended from day 3 to day 12. The leukopenia may be because of the immunosuppressive effect of chemotherapeutic drugs^52^. Also, there was a significant increase in the total number of lymphocytes and monocytes and decreased neutrophils after treating EAC-bearing mice with Cis or CF NPs. This increase could be because of the stress of EAC-cells^53^. The platelets count decreased in EAC-bearing mice; however, after treatment with Cis or CF, NPs restored close to their normal values. This reduction in platelet count in EAC-bearing mice could be because of the suppressive effect of EAC toxin on the bone marrow^54,55^. Inoculation with EAC for 14 days increased the activities of ALT, AST, and the levels of urea and creatinine. The treatment of EAC-bearing mice with Cis or CF NPs decreased these parameters compared with EAC-bearing mice alone. The increase in the previous biochemical parameters could be because of EAC cell hepatic and kidney dysfunction caused by liver and kidney injury^56^. The present study showed prominent histopathological alterations in the liver of EAC-bearing mice. These changes appear as marked disorganization of the hepatic architecture, and mostly hepatocytes were vacuolated; others with pyknotic and karyolitic nuclei, and irregular blood sinusoids with distinct Kupffer cells were observed. These findings agreed with those of^57^, who reported that loading mice by EAC deteriorate lobular architecture where hepatocytes showed enlarged nuclei, hydropic degeneration, and sinusoidal infiltration of carcinoma cells mixed with lymphocytes. The liver sections of EAC-bearing mice treated with Cis exhibited slight disorganization of hepatic architecture, irregular strands of hepatocytes with normal stainability, and dilated blood sinusoids with activated Kupffer cells. Such findings were parallel with^58^, who stated that hepatotoxicity and nephrotoxicity are caused by cisplatin. Treatment of EAC-bearing mice with CF NPs revealed substantial improvement in hepatic cellularity; hepatocytes had kept their regular shape with their basophilic cytoplasm and large centric rounded nuclei, slightly widening blood sinusoids with phagocytic Kupffer cells were found. These results were consistent with a previous study by^59^, who reported that the histopathological results confirmed kidney damage in EAC-bearing mice, which revealed the disorganization of the kidney tissue, damage of mostly renal tubules. These results were supported by^57^, who stated that loading mice by EAC showed marked degenerative changes in renal tubules, stromal congestion, and moderate inflammation with mild degeneration of glomeruli. Kidney section of EAC-bearing mice treated with Cis exhibited slight disorganization of the renal structure, normal like glomeruli, and few renal tubules were destroyed. These results agreed with^58^. EAC-bearing mice treated with CF NPs exhibited partial improvement that appeared as normal, like glomeruli with regular Bowman’s space, and mostly renal tubules are normal; others are damaged. These results were similar to those of^59^.

## Conclusion

In conclusion, the effect of Zn^2+^ ions doping on the structural and morphological properties of Co_1−x_Zn_x_Fe_2_O_4_ (x = 0,0.35) nanoparticles were investigated. Two nanosized cobalt ferrite samples with varying zinc contents were prepared using the auto-combustion flash method route. The morphology of the synthesized nano-ferrite is almost spherical with certain distortion which may be due to doping. The FTIR spectrum inveterate a spinel phase formation of having ions distributed in the octahedral and tetrahedral sites. Also, this study reported that CF NPs had in vitro and in vivo anticancer effects and improved the hematological, biochemical, and histopathological alterations induced by EAC- inoculation in mice.

## Data Availability

All data generated or analyzed during this study are included in this published article. More detailed data is available from the corresponding author on reasonable request.

## References

[1] World Health Organization (WHO) 2020: Cancer.

[2] Sung, H. et al. Global cancer statistics 2020: GLOBOCAN estimates of incidence and mortality worldwide for 36 Cancers in 185 Countries. *CA Cancer J. Clin.* **71**(3), 209–249 (2021). 33538338 10.3322/caac.21660

[3] Bhupendra, K. *Types of cancer herbs for cancer treatment* 53–150 (2020).

[4] Schirrmacher, V. From chemotherapy to biological therapy: A review of novel concepts to reduce the side effects of systemic cancer treatment (review). *Int. J. Oncol.* **54**(2), 407–419 (2019). 30570109 10.3892/ijo.2018.4661 PMC6317661

[5] El-Naggar, S. A., Abdel-Farid, I. B., Germoush, M. O., Elgebaly, H. A. & Alm-Eldeen, A. A. Efficacy of *Rosmarinus officinalis* leaves extract against cyclophosphamide-induced hepatotoxicity. *Pharm. Biol.* **54**(10), 2007–2016 (2016). 26828825 10.3109/13880209.2015.1137954

[6] El-Naggar, A. E., El-Gowilly, S. M. & Sharabi, F. M. Possible ameliorative effect of ivabradine on the autonomic and left ventricular dysfunction induced by doxorubicin in male rats. *J. Cardiovasc. Pharmacol.* **72**(1), 22–31 (2018). 29688913 10.1097/FJC.0000000000000586

[7] El-Naggar, S. A., El-Said, K. S., Mobasher, M. & Elbakry, M. Enhancing antitumor efficacy of cisplatin low dose by EDTA in Ehrlich ascetic carcinoma bearing mice. *Braz. Arch. Biol. Technol.* **62**, e19180716 (2019).

[8] Nessa, M. U., Rahman, M. & Kabir, Y. Plant-produced monoclonal antibody as immunotherapy for cancer. *BioMed. Res. Int.* **2020**, 3038564 (2020). 10.1155/2020/3038564 PMC7468595 32908881

[9] Hola, K., Markova, Z., Zoppellaro, G., Tucek, J. & Zboril, R. Tailored functionalization of iron oxide nanoparticles for MRI, drug delivery, magnetic separation and immobilization of biosubstances. *Biotechnol. Adv.* **33**(6 Pt 2), 1162–1176 (2015). 25689073 10.1016/j.biotechadv.2015.02.003

[10] El-Deeb, N. M., El-Sherbiny, I. M., El-Aassar, M. R. & Hafez, E. E. Novel trend in colon cancer therapy using silver nanoparticles synthesized by honey bee. *Nanomed. Nanatechnol.* **6**(2), 265 (2015).

[11] Bromma, K. & Chithrani, B. D. Advances in gold nanoparticle-based combined cancer therapy. *J. Nanomater*. **10**(9), 1671 (2020). 10.3390/nano10091671 PMC7557687 32858957

[12] Tarantash, M., Nosrati, H., KheiriManjili, H. & Baradar Khoshfetrat, A. Preparation, characterization and in vitro anticancer activity of paclitaxel conjugated magnetic nanoparticles. *Drug Dev. Ind. Pharm.* **44**, 1895–1903 (2018). 30073853 10.1080/03639045.2018.1508222

[13] Jia, Y. et al. Co-encapsulation of magnetic Fe3O4 nano¬particles and doxorubicin into biodegradable PLGA nanocarriers for intratumoral drug delivery. *Int. J. Nanomed.* **63**, 1697–1708 (2012). 10.2147/IJN.S28629 PMC3356178 22619520

[14] Weissleder, R. et al. Ultrasmall superparamagnetic iron oxide: An intravenous contrast agent for assessing lymph nodes with MR imaging. *Radiology* **175**(2), 494–498 (1990). 2326475 10.1148/radiology.175.2.2326475

[15] Akhtar, M. F. et al. A comprehensive review on the applications of ferrite nanoparticles in the diagnosis and treatment of breast cancer. *Med. Oncol.* **41**(2):53. 10.1007/s12032-023-02277-2 (2024). 10.1007/s12032-023-02277-2 38198041

[16] Samia Dhahri, Y. et al. Morphological and nanomechanical insights into the selective targeting of breast cancer cells by novel nickel ferrite nanoparticles. *Sci. Rep.*10.1038/s41598-026-44134-y (2026). 41997973 10.1038/s41598-026-44134-y PMC13247270

[17] Alahmari, F., Jaremko, M., Khan, F. A., Sozeri, H. & Sertkol, M. Electrospun Cu–Co ferrite nanofibers: Synthesis, structure, optical and magnetic properties, and anti-cancer activity. *RSC Adv.* **14**, 7540–7550. 10.1039/d3ra08087k (2024). 10.1039/d3ra08087k PMC10910578 38440265

[18] Çelik, M., Kucuk, I., Sadak, S. & Uslu, B. Advanced ferrite hybrid nanostructures for electrochemical sensing: a review of biomedical diagnostic applications. *Microchem. J.* **218**, 115507. 10.1016/j.microc.2025.115507 (2025).

[19] Narayanaswamy, V., Rah, B., Muhammad, J. S., Al-Omari, I. A., Kamzin, A. S., Obaidat, I. M. & Issa, B. Evaluation of antiproliferative properties of CoMnZn-Fe_2_O_4_ ferrite nanoparticles in colorectal cancer cells. Pharmaceuticals **17**, 327. 10.3390/ph17030327 (2024). 10.3390/ph17030327 PMC10973991 38543113

[20] Shen, Y. & TanTai, J. Co-delivery anticancer drug nanoparticles for synergistic therapy against lung cancer cells. *Drug Des. Devel Ther.* **23**(14), 4503–4510 (2020). 10.2147/DDDT.S275123 PMC7591005 33122893

[21] Ghasem, M., Seyed, S., Ehsan, K. & Tabrizi Masoud. The cytotoxic properties of zinc oxide nanoparticles on the rat liver and spleen, and its anticancer impacts on human liver cancer cell lines. *J. Biochem. Mol. Toxi*. **33**(7), e22324 (2019). 10.1002/jbt.22324 30951608

[22] Bohara, R. A., Thorat, N. D., Yadav, H. M. & Pawar, S. H. One-step synthesis of uniform and biocompatible amine functionalized cobalt ferrite nanoparticles: A potential carrier for biomedical applications. *New. J. Chem.* **38**(7), 2979–2986 (2014).

[23] Li, J., Ng, D. H., Song, P., Song, Y. & Kong, C. Bio-inspired synthesis and characterization of mesoporous ZnFe_2_O_4_ hollow fibers with enhancement of adsorption capacity for acid dye. *J. Ind. Eng. Chem.* **23**, 290–298 (2015).

[24] Wang, L., Gulati, P., Santra, D., Rose, D. & Zhang, Y. Nanoparticles prepared by prosomillet protein as novel curcumin delivery system. *Food Chem.* **240**, 1039–1046 (2018). 28946220 10.1016/j.foodchem.2017.08.036

[25] Sun, B., Tian, Y., Chen, L. & Jin, Z. Linear dextrin as curcumin delivery system: Effect of degree of polymerization on the functional stability of curcumin. *Food Hydrocoll.* **77**, 911–920 (2018).

[26] Salih, S. J. & Mahmood, W. M. Review on magnetic spinel ferrite (MFe_2_O_4_) nanoparticles: From synthesis to application. *Heliyon* **9**(6), e16601. 10.1016/j.heliyon.2023.e16601 (2023). 10.1016/j.heliyon.2023.e16601 PMC10238938 37274649

[27] Ghasemian, G. S. D., Abdolahi, Z. & Dana, M. In vitro evaluation of cobalt-zinc ferrite nanoparticles coated with DMSA on human prostate cancer cells. *J. Mol. Biomarkers Diagnosis*. **4**(3), 1000154 (2013).

[28] Al-Qubaisi, M. S. et al. Cytotoxicity of nickel zinc ferrite nanoparticles on cancer cells of epithelial origin. *Int. J. Nanomed.* **8**, 1–12 (2013). 10.2147/IJN.S42367 PMC3716602 23885175

[29] Vita, P. et al. Accumulation and biological effects of cobalt ferrite nanoparticles in human pancreatic and ovarian cancer cells. *Medicina* **50**, 237–244 (2014). 10.1016/j.medici.2014.09.009 25458961

[30] Mansour, S. F., Hemeda, O. M., El-Dek, S. I. & Salem, B. I. Influence of La doping and synthesis method on the properties of CoFe_2_O_4_ nanocrystals. *J. Magn. Mater. Magn.* **420**, 7–18 (2016).

[31] Weir, N. M. et al. Curcumin induces G2/M arrest and apoptosis in cisplatin-resistant human ovarian cancer cells by modulating Akt and p38 MAPK. *Cancer Biol. Ther.* **61**, 78–184 (2007). 10.4161/cbt.6.2.3577 PMC1852522 17218783

[32] Huang, K. H., Li, X., Ravindran, V. & Bryden, W. L. Comparison of apparent ileal amino acid digestibility of feed ingredients measured with broilers, layers, and roosters. *Poult. Sci.* **85**(4), 625–634 (2006). 16615346 10.1093/ps/85.4.625

[33] Fawcett, J. K. & Scott, J. E. A rapid and precise method for the determination of urea. *J. Clin. Pathol.* **13**(2), 156–159 (1960). 13821779 10.1136/jcp.13.2.156 PMC480024

[34] Bancroft, J. D. & Gamble, M. *Theory and Practice of Histological Techniques* 6th edn, 725 (Churchill Livingstone/Elsevier, 2008).

[35] Thomas, M. & George, K. C. Infrared and magnetic study of nanophase zinc ferrite. *Indian J. Pure Ap Phy*. **47**, 81–86 (2009).

[36] Sathishkumar, G., Venkataraju, C. & Sivakumar, K. Effect of nickel on the structural and magnetic properties of nano structured CoZnFe_2_O_4_. *J. Mater. Sci: Mater. Electron*. **22**, 1715–1724 (2011).

[37] Asogekar, P., Gaonkar, S., Kumar, A. & Verenkar, V. Influence of Co over magnetically benign Zn ferrite system and study of its structural, dielectric, superparamagnetic and antibacterial efficacy. *Mater. Res. Bull.*, **141**, 111330 (2021).

[38] Manohar, A., Krishnamoorthi, C., Naidu, K. & Pavithra, C. Dielectric, magnetic hyperthermia and photocatalytic properties of ZnFe_2_O_4_ nanoparticles synthesized by solvothermal reflux method. *Appl. Phys. A: Mater. Sci. Process.*, **125**, 477 (2019).

[39] Ghosh, M., Sonkar, R., Phukan, G., Borahc, J. & Chowdhury, D. Cobalt ion-incorporated nanocrystalline spinel cubic zinc ferrite for targeted magnetic hyperthermia and sensing applications. *RSC Adv.* **15**, 12964 (2025). 40271408 10.1039/d5ra01897h PMC12013603

[40] Annie, V., Xavier, B., Krishnan, S. & Jerome Das, S. Investigation on the magnetically separable Zn substituted CoFe_2_O_4_ nanoparticles with enhanced photo-fenton degradation. *J. Nanosci. Nanotechnol*. **18**(8), 5354–5366 (2018). 29458587 10.1166/jnn.2018.15427

[41] Vinosha, P. et al. Review on recent advances of zinc substituted cobalt ferrite nanoparticles: Synthesis characterization and diverse applications. *Ceram. Int.* **47**, 10512–10535. (2021).

[42] El-Nahass, E., Salem, B., El-Naggar, S. & Elwan, M. Evaluation the toxic effects of cobalt–zinc ferrite nanoparticles in experimental mice. *Sci. Rep.* **15**, 6903 (2025). 40011491 10.1038/s41598-025-90043-x PMC11865507

[43] Mondal, N., Sonkar, R., Boro, B., Ghosh, M. & Chowdhury, D. Nanocrystalline Ni–Zn spinel ferrites: size-dependent physical, photocatalytic and antioxidant properties. *Nanoscale Adv.*, **5**, 5460–5475. (2023). 10.1039/d3na00446e PMC10563843 37822912

[44] Pradeep, A. & Chandrasekaran, G. FTIR study of Ni, Cu and Zn substituted nano-particles of MgFe_2_O_4_. *Mater. Lett.* **60**(3), 371–374 (2006).

[45] Mostafa, M. & Salem, B. Studying structural, molecular, morphological and electrical properties of Co_0.2_Zn_0.8_Fe_2_O_4_ doped with Cadmium. *Mater. Sci. Eng. B*. **286**, 116043 (2022).

[46] Anselmo, A. C. & Mitragotri, S. Nanoparticles in the clinic. *Bioeng. Transl. Med.* **1**(1), 10–29 (2016). 29313004 10.1002/btm2.10003 PMC5689513

[47] Moghaddam Boroumand, A. et al. Eco-friendly formulated zinc oxide nanoparticles: Induction of cell cycle arrest and apoptosis in the MCF-7 cancer cell line. *Genes (Basel)*. **8**(10), 281 (2017). 29053567 10.3390/genes8100281 PMC5664131

[48] Fatoh, A. E. et al. Cytotoxic impact of zinc oxide nanoparticles against Ehrlich ascites carcinoma cells in mice. *Int. J. Pharm. Sci.* **4**(3), 560–564 (2014).

[49] Sasaki, D. et al. Development of nanoparticles derived from corn as mass producible bionanoparticles with anticancer activity. *Sci. Rep.* **11**, 22818 (2021). 34819568 10.1038/s41598-021-02241-y PMC8613273

[50] Zuo, W. et al. Synthesis and application of Au NPs-chitosan nanocomposite in the treatment of acute myeloid leukemia in vitro and in vivo. *Arab. J. Chem.* **14**(2), 102929 (2021).

[51] Salem, M. L. et al. Kinetics of rebounding of lymphoid and myeloid cells in mouse peripheral blood, spleen and bone marrow after treatment with cyclophosphamide. *Cell. Immunol.* **276**(1–2), 67–74 (2012). 22560674 10.1016/j.cellimm.2012.03.010 PMC3787597

[52] Tian, J. et al. Branched-chain amino acids catabolism pathway regulation plays a critical role in the improvement of leukopenia induced by cyclophosphamide in 4T1 tumor bearing mice treated with lvjiaobuxue granule. *Front. Pharmacol.* **12**, 657047 (2021). 34759816 10.3389/fphar.2021.657047 PMC8573099

[53] El-Bolkiny, Y. E., Salem, M. L., El-Naggar, S. A. & El-Sharkawy, F. R. Ameliorating effects of rosemary and costus on blood-associated toxicity in Ehrlich-bearing mice treated with cisplatin. *Int. J. Cancer BioMed. Res.* **5**(4), 85–98 (2021).

[54] Steensma, D. P. Is anemia of cancer different from chemotherapy induced anemia? *J. Clin. Oncol.* **26**(7), 1022–1024 (2008). 18227523 10.1200/JCO.2007.15.3874

[55] Kumar, R. S., Rajkapoor, B. & Perumal, P. vitro and in vivo anticancer activity of Indigofera cassioides Rottl. Ex. *DC Asian Pac. J. Trop. Med.* **4**(5), 379–385 (2011). 21771681 10.1016/S1995-7645(11)60108-9

[56] Ali, D. A., Badr El-Din, N. K. & Abou-El-magd, R. F. Antioxidant and hepatoprotective activities of grape seeds and skin against Ehrlich solid tumor induced oxidative stress in mice. *Egypt. J. Basic. Appl. Sci.* **2**(2), 98–109 (2015).

[57] Ahmed, S. H., Eissa, D. M., Hassan, M. K. & Abaas, O. A. Ameliorativeeffect of the Aurelia aurita crude venom on the murine ehrlish ascites carcinoma-induced hepatotoxicity and nephrotoxicity. *Int. J. Adv. Res.* **5**(1), 1167–1178 (2017).

[58] El-Naggar, S. A., Alm-Eldeen, A. A., Mousa, O., El-Boray, K. F. & Elgebaly, H. A. Ameliorative effect of propolis against cyclophosphamide-induced toxicity in mice. *Pharm. Biol.* **53**(2), 235–241 (2015). 25289525 10.3109/13880209.2014.914230

[59] Ibrahim, M. A., Bakhaat, G. A., Tammam, H. G., Mohamed, R. M. & El-Naggar, S. A. Cardioprotective effect of green tea extract and vitamin E on Cisplatininduced cardiotoxicity in mice: Toxicological, histological and immunohistochemical studies. *Biomed. Pharmacother*. **113**, 108731 (2019). 30851549 10.1016/j.biopha.2019.108731

